# Myomaker, Regulated by MYOD, MYOG and miR-140-3p, Promotes Chicken Myoblast Fusion

**DOI:** 10.3390/ijms161125946

**Published:** 2015-11-02

**Authors:** Wen Luo, Erxin Li, Qinghua Nie, Xiquan Zhang

**Affiliations:** 1 Department of Animal Genetics, Breeding and Reproduction, College of Animal Science, South China Agricultural University, Guangzhou 510642, China; lw729@stu.scau.edu.cn (W.L.); erxin.li@cix.csi.cuny.edu (E.L.); nqinghua@scau.edu.cn (Q.N.); 2 Guangdong Provincial Key Lab of Agro-Animal Genomics and Molecular Breeding, South China Agricultural University, Guangzhou 510642, China; 3 Key Lab of Chicken Genetics, Breeding and Reproduction, Ministry of Agriculture, South China Agricultural University, Guangzhou 510642, China

**Keywords:** chicken, Myomaker, myoblast fusion, MYOD, MYOG, miR-140-3p

## Abstract

The fusion of myoblasts is an important step during skeletal muscle differentiation. A recent study in mice found that a transmembrane protein called Myomaker, which is specifically expressed in muscle, is critical for myoblast fusion. However, the cellular mechanism of its roles and the regulatory mechanism of its expression remain unclear. Chicken not only plays an important role in meat production but is also an ideal model organism for muscle development research. Here, we report that Myomaker is also essential for chicken myoblast fusion. Forced expression of *Myomaker* in chicken primary myoblasts promotes myoblast fusion, whereas knockdown of *Myomaker* by siRNA inhibits myoblast fusion. MYOD and MYOG, which belong to the family of myogenic regulatory factors, can bind to a conserved E-box located proximal to the *Myomaker* transcription start site and induce *Myomaker* transcription. Additionally, miR-140-3p can inhibit *Myomaker* expression and myoblast fusion, at least in part, by binding to the 3ʹ UTR of *Myomaker in vitro*. These findings confirm the essential roles of *Myomaker* in avian myoblast fusion and show that MYOD, MYOG and miR-140-3p can regulate *Myomaker* expression.

## 1. Introduction

Skeletal muscle, which composes approximately half of total body mass, is an important tissue involved in regulating the metabolism, locomotion and strength of the animal body [1]. The formation of skeletal muscle requires the mononucleated myoblasts to withdraw from the cell cycle and to fuse with each other to form nascent, multinucleated myotubes. Then, the nascent myotubes undergo further cell fusion and express contractile proteins to form mature myotubes. The fusion of myoblasts is a fundamental step during muscle differentiation, and this step involves several cellular and molecular behaviours, such as cell migration, recognition, adhesion, membrane alignment, signalling transduction and actin cytoskeletal reorganization, leading up to the final membrane fusion [2]. Many of these cellular and molecular events are conserved in vertebrates [3]; therefore, studies in flies, zebrafish, mice and other vertebrate model systems have provided a clearer understanding about these events [3,4].

Myoblast fusion is a highly regulated process. Recent advances in this field have revealed many molecules and signalling pathways that are involved in this process [3]. Among these regulatory molecules, transmembrane proteins, which are a type of membrane protein that spans the entirety of the biological membrane, play important roles during myoblast fusion. Many cellular events, such as cell migration, recognition and adhesion, require this type of protein to complete the fusion process. Myoferlin, a transmembrane protein that is expressed at apposed membranes sites undergoing fusion, can bind to phospholipids in a calcium-sensitive manner [5]. A mutation in myoferlin C2A can disrupt this binding and decrease the fusion efficiency of large myotubes [5]. As a type I transmembrane protein, the mannose receptor is also required for the fusion of myoblasts due to its role in directing myoblast migration [6]. In *Drosophila*, direct evidence suggests that the fusion of myoblasts are dependent on transmembrane proteins of the immunoglobulin superfamily, which include Kin of IrreC/Dumbfounded (Kirre/Duf) [7], Roughest/Irregular-optic chiasma C (Rst/Irre-C) [8], Hibris (Hbs) and Sticks-and-stones (Sns) [9,10]. However, none of the above proteins is muscle-specific, and many of them have redundant roles during myoblast fusion. Therefore, a muscle-specific transmembrane protein with a direct and essential role in myoblast fusion remains an attractive target for discovery.

Recently, the muscle-specific transmembrane protein transmembrane protein 8c (Tmem8c), also called Myomaker, was found to be necessary for myoblast fusion [11]. During myogenesis and muscle regeneration, Myomaker is expressed transiently and promotes myoblast fusion efficiently [11,12]. *Myomaker* genetic disruption in mice not only completely abolishes muscle regeneration by adult satellite cells but also causes perinatal death of embryos due to a complete block of myoblast fusion. The protein sequence of Myomaker is highly conserved across vertebrate organisms [11], and its function in myogenesis is conserved between mice and zebrafish [13]. However, the expression pattern and function of Myomaker in avian myogenesis have not been explored, and the cellular mechanism of its function and the regulatory mechanism of its expression during myogenesis remain to be determined. MYOG and MYOD are critical transcription factors in myogenesis and can regulate the transcription of most of the muscle-specific genes [14,15,16,17]. Both of them play an important role in the regulation of myoblast differentiation. *MYOD* act as a myogenic determination gene [15], whereas *MYOG* is essential for the terminal differentiation of committed myoblasts [17]. Here, we found the regulatory role of MYOG and MYOD in the transcription of *Myomaker*, and report the expression pattern of these genes during chicken embryonic skeletal muscle development and the differentiation of primary myoblast. Myomaker function in chicken myoblast fusion was explored by overexpression and loss-of-function assays. In addition, we analysed the mRNA expression patterns of *MYOD*, *MYOG* and *Myomaker* and found that MYOD and MYOG can bind directly to the promoter of *Myomaker* and induce its transcription during myoblast fusion. Finally, to understand the post-transcriptional regulation of *Myomaker* expression, we analysed the 3ʹ UTR of *Myomaker* and found that miR-140-3p can inhibit *Myomaker* expression by binding to the *Myomaker* 3ʹ UTR *in vitro*. miR-140-3p overexpression inhibited the late stage of myoblast differentiation but promoted myoblast proliferation. Collectively, our results confirmed the important roles of Myomaker in avian myoblast fusion and found that MYOD, MYOG and miR-140-3p could regulate *Myomaker* expression.

## 2. Results

### 2.1. cDNA Sequence, Genomic Structure and Protein Conservation of the Chicken Myomaker Gene

**Figure 1:**
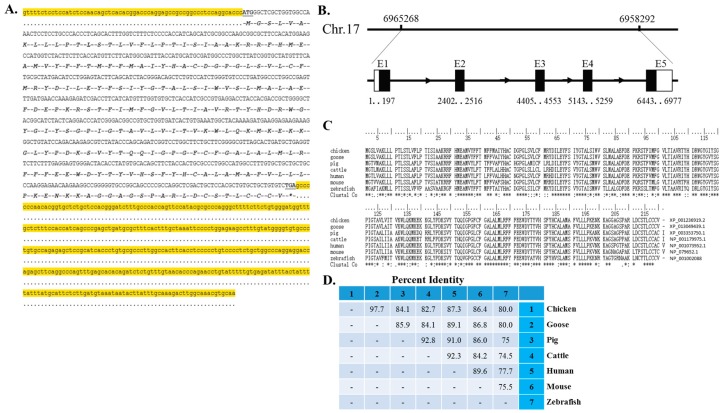
cDNA sequence, genomic structure and protein conservation of the chicken Myomaker gene. (**A**) The obtained cDNA sequence of chicken *Myomaker* transcripts. Nucleotides highlighted in yellow represent the 5ʹ UTR and 3ʹ UTR. Nucleotides in capital letters represent open reading frames, and letters below represent encoded amino acids. * represents stop codon; (**B**) Genomic structure of the chicken *Myomaker* gene. Black boxes indicate coding sequence regions, and white boxes indicate UTRs; (**C**) Amino acid alignment of Myomaker proteins from chicken, goose, pig, cattle, human, mouse and zebrafish. Conserved sequences are marked with asterisk within the line of Clustal Co.; (**D**) Percent identities of Myomaker amino acids compared to chicken, goose, pig, cattle, human, mouse and zebrafish Myomaker amino acids.

To begin to study the *Myomaker* gene in chicken, we first isolated its full-length cDNA and analysed its genomic structure and protein conservation. The obtained cDNA of chicken *Myomaker* gene was 1113 bp in length with a 62 bp 5ʹ UTR, a 663 bp open reading frame, and a 388 bp 3ʹ UTR (Figure 1A, accession number of KP230536 in the NCBI database). The gene is located at 6,958,292–6,965,268 nucleotide of chicken chromosome 17 (GGA 17) and spans 6977 bp containing five exons and four introns (Figure 1B). Amino acid alignment of Myomaker proteins from chicken, goose, pig, cattle, human, mouse and zebrafish shows strong conservation (Figure 1C), indicating its conserved function among vertebrates. Blast search results showed that the percent identities of the chicken Myomaker protein were 97.7%, 84.1%, 82.7%, 87.3%, 86.4% and 80.0% compared to those of goose, pig, cattle, human, mouse and zebrafish, respectively (Figure 1D).

### 2.2. Myomaker mRNA Expression during Chicken Skeletal Muscle Development

**Figure 2:**
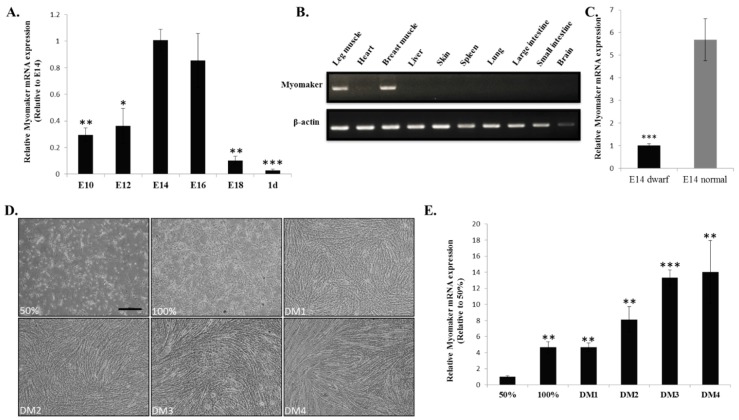
*Myomaker* mRNA expression during chicken skeletal muscle development. **(A)** The relative mRNA expression of *Myomaker* in chicken embryonic leg muscle. β-Actin was used as the reference gene; (**B**) RT-PCR for detecting *Myomaker* mRNA expression in 10 tissues from E14 chicken. The upper panel shows the bands of *Myomaker* mRNA; the lower panel shows the bands of *β-actin* mRNA, which was used as the reference gene. All cropped gels have been run under the same experimental conditions; (**C**) The relative mRNA expression of *Myomaker* between E14 leg muscles of dwarf chickens and normal chickens; (**D**) Phase-contrast micrographs of proliferating (GM, 50% and 100% confluency) and differentiating (DM) chicken primary myoblasts. Bar, 50 µm; (**E**) The relative mRNA expression of *Myomaker* during chicken primary myoblast differentiation. The data in A and C are mean ± S.E.M. with six chickens per group. The data in E are mean ± S.E.M. with four cultures per group. One-sample *t* test was used to assess the change from each data point to the control (E14 in A and 50% in E). * *p* < 0.05; ** *p* < 0.01; *** *p* < 0.001.

A gene expression pattern often correlates with its function. To investigate the potential involvement of Myomaker in chicken myoblast fusion, we examined its expression profile during embryonic skeletal muscle development and primary myoblast differentiation. During skeletal muscle development in embryonic chicken, *Myomaker* mRNA expression is up-regulated from embryonic day 10 (E10) to E14 and sharply down-regulated after E16 (Figure 2A). Among these embryonic days, E14 and E16 showed the highest expression of *Myomaker*, suggesting that potential active cell fusion events occurred during these embryonic days in chicken. Similar to the skeletal muscle-specific expression of *Myomaker* mRNA in mice [11], RT-PCR of *Myomaker* in E14 chicken embryo also indicated specific expression in skeletal muscle (Figure 2B). Additionally, *Myomaker* mRNA expressed higher level in E14 skeletal muscle of normal chickens than in that of dwarf chickens (Figure 2C), suggesting that fast-growing chickens have more abundant *Myomaker* mRNA expression than slow-growing chickens.

To further study the expression pattern of *Myomaker in vitro*, we separated chicken primary myoblasts and induced them to differentiate (Figure 2D). During myoblast differentiation and fusion, *Myomaker* mRNA expression increased (Figure 2E). These results demonstrate that *Myomaker* expression is skeletal muscle-specific and is transiently up-regulated during myoblast fusion in chickens.

### 2.3. Myomaker Is Essential for Myoblast Fusion in Chicken

**Figure 3:**
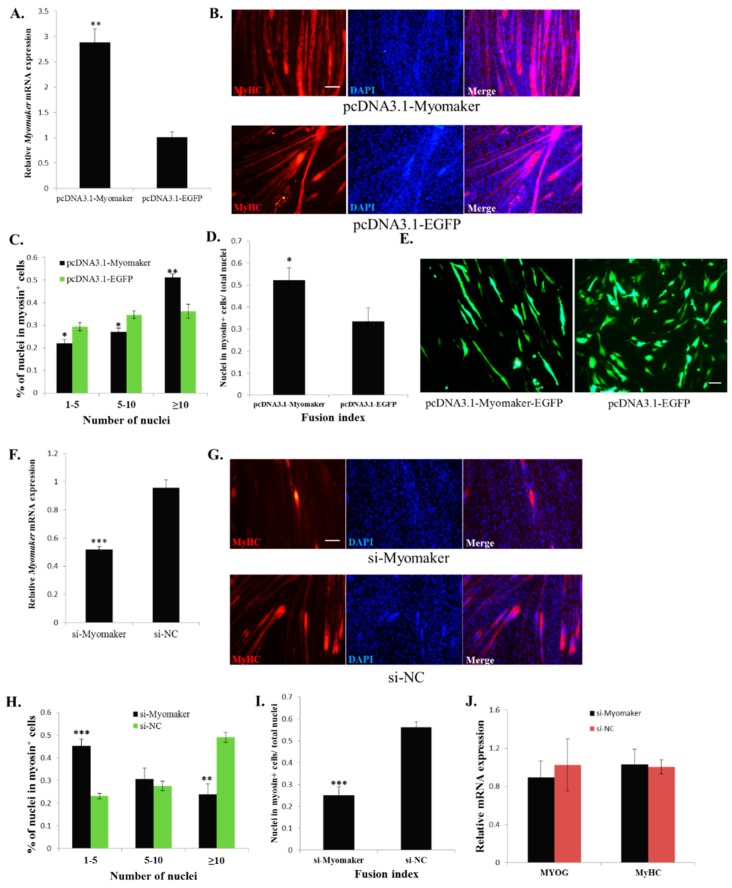
Myomaker is essential for myoblast fusion in chicken. (**A**) *Myomaker* overexpression via pcDNA3.1-Myomaker transfection significantly increased the *Myomaker* mRNA level; (**B**) MyHC immunostaining of primary myoblasts transduced with pcDNA3.1-Myomaker or pcDNA3.1-EGFP and differentiated for 48 h. Bar, 100 µm; (**C**) The distribution of myonuclear content in the cells with over-expressed *Myomaker* or *EGFP* (**D**) The fusion index of the cells transfected with pcDNA3.1-Myomaker or pcDNA3.1-EGFP; (**E**) Fluorescence images of myoblasts transfected with pcDNA3.1-Myomaker-EGFP or pcDNA3.1-EGFP at 36 h. Bar, 50 µm; (**F**) *Myomaker* knockdown via si-Myomaker transfection significantly reduced the *Myomaker* mRNA level; (**G**) MyHC immunostained of primary myoblasts transduced with si-Myomaker or si-NC and differentiated for 48 h. The nuclei were visualized with DAPI. Bar, 100 µm; (**H**) The distribution of myonuclear content in the cells with knockdown *Myomaker* or NC; (**I**) The fusion index of the cells transfected with si-Myomaker and si-NC; (**J**) *MYOG* and *MyHC* mRNA expression was not influenced by *Myomaker* knockdown. The data in A, F and J are mean ± S.E.M. with four cultures per group. The data in C, D, H and I are mean ± S.E.M. with three cultures per group. One-sample *t* test was used to assess the change between the two groups. * *p* < 0.05; ** *p* < 0.01; *** *p* < 0.001.

To study the function of *Myomaker* in chicken myoblast fusion, we first overexpressed *Myomaker* during myoblast differentiation and observed its effect on myoblast fusion. The results showed that *Myomaker* mRNA was elevated approximately three-fold after 48 h of pcDNA3.1-Myomaker transfection (Figure 3A). *Myomaker* overexpression in primary myoblasts promoted cell fusion and generated larger myotubes with a greater number of nuclei than the control cells (Figure 3B,C). Additionally, the fusion index of Myomaker-transfected cells significantly increased (Figure 3D). To identify the behaviour of Myomaker-overexpressing myoblasts specifically, Myomaker-EGFP fusion protein overexpression plasmid was constructed and transfected into the myoblasts and then switched to differentiation media. The fluorescence of EGFP was then used as a tracer to assess the fusion of *Myomaker*-overexpressing myoblasts. The results showed that many of the *Myomaker*-overexpressing cells were fused into long myotubes, whereas the control cells remained single nuclei (Figure 3E), suggesting that *Myomaker* overexpression promotes myoblast fusion. In addition, we also introduced si-Myomaker, designed specifically for *Myomaker*, into primary myoblasts to investigate the effect of *Myomaker* loss-of-function. Cells transfected with si-Myomaker showed reduced expression of *Myomaker* (Figure 3F), and lead to fewer and smaller myotubes with fewer nuclei than the negative control cells (Figure 3G,H). Additionally, the fusion index of si-Myomaker transfected cells significantly decreased (Figure 3I). However, the differentiation markers *MYOG* and *MyHC* were expressed normally in *Myomaker* knockdown cells, suggesting that *Myomaker* inhibition specifically influenced myoblast fusion (Figure 3J). Therefore, the above results demonstrate that *Myomaker* plays an essential role in chicken myoblast fusion.

### 2.4. Myomaker Transcription Is Controlled by a Conserved Promoter E-Box Element in Chicken

**Figure 4:**
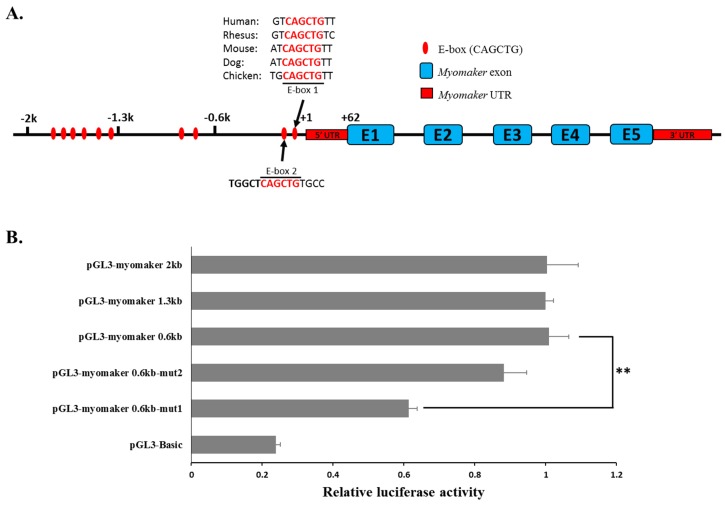
*Myomaker* transcription is controlled by a conserved promoter E-box element in chicken. (**A**) The location of the ten E-boxes in the 2-kb upstream region of the *Myomaker* TSS; (**B**) Identification of the core region in the *Myomaker* promoter by luciferase reporter assays. The 0.6-kb, 1.3-kb and 2.0-kb upstream regions of the *Myomaker* TSS were inserted into the pGL3 plasmid and then transfected into the established myotubes. Luciferase activities were measured after 36 h transfection. The data in B are mean ± S.E.M. with four cultures per group. One-way ANOVA with a Bonferroni post-hoc tests was used to analyze the data. ** *p* < 0.01.

To define the mechanism that controls the transcription of chicken *Myomaker*, we analysed its 5ʹ flanking region and searched for the core region that determines *Myomaker* promoter activity. Three fragments, including 2-kb, 1.3-kb and 0.6-kb upstream regions of the *Myomaker* transcription start site (TSS) (Figure 4A), were amplified and cloned into the pGL3-basic vector and transfected into the established myotubes. The results showed that these three regions have the same promoter activity (Figure 4B), suggesting that the shortest fragment, at 0.6 kb upstream, contained the core region. A previous study in mice showed that two E-box elements in the promoter region of *Myomaker* control gene transcription [12]. In chickens, 10 E-box elements were found in the 2-kb upstream region of the *Myomaker* TSS. Among these 10 E-boxes, only two are located in the 0.6-kb upstream fragment (Figure 4A), and E-box 1 is conserved among vertebrates. We mutated E-box 1 and E-box 2 and found that the mutation of E-box 1 significantly decreased promoter activity, whereas the mutation of E-box 2 had no significant impact on promoter activity (Figure 4B). Therefore, E-box 1 in the *Myomaker* promoter is essential for its transcription.

### 2.5. MYOD and MYOG Bind to the E-Box 1 Region and Regulate Myomaker Transcription

**Figure 5:**
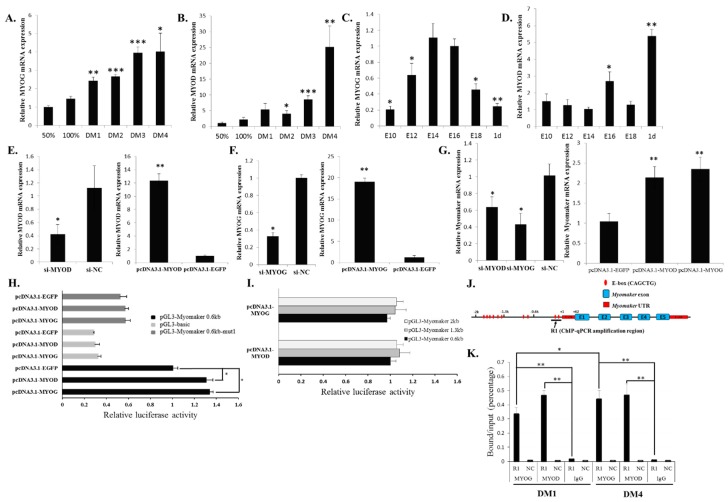
MYOD and MYOG can bind to E-box 1 and control *Myomaker* transcription. (**A**) *MYOG* mRNA expression during myoblast differentiation; (**B**) *MYOD* mRNA expression during myoblast differentiation; (**C**) *MYOG* mRNA expression in stages of chicken embryonic leg muscle development; (**D**) *MYOD* mRNA expression in stages of chicken embryonic leg muscle development; (**E**) *MYOG* mRNA expression after pcDNA3.1-MYOG and si-MYOG transfection; (**F**) *MYOD* mRNA expression after pcDNA3.1-MYOD and si-MYOD transfection; (**G**) *Myomaker* mRNA expression after the transfection of siRNA and *MYOG* and *MYOD* overexpression vectors; (**H**) *MYOG* or *MYOD* overexpression in myoblasts can significantly increase the luciferase activity of pGL3-Myomaker 0.6 kb but cannot influence the luciferase activity of E-box 1 mutated pGL3-Myomaker 0.6 kb-mut1; (**I**) *MYOG* or *MYOD* overexpression in myoblasts cannot enhance the luciferase activity of pGL3-Myomaker 1.3 kb and pGL3-*Myomaker* 2 kb compared to that of pGL3-Myomaker 0.6 kb. The relative luciferase activities have been normalized to the promoter activity that transfected pcDNA3.1-EGFP in each condition; (**J**) Schematic representation of the ChIP-qPCR amplification region (indicated by arrow); (**K**) ChIP-qPCR analysis using anti-MYOG, anti-MYOD or chicken IgG showed that MYOG and MYOD could bind to the R1 region of the chicken *Myomaker* gene in myoblasts at DM1 and DM4. A region from the *GAPDH* gene was amplified as a negative control to verify the specificity of the enrichment (showed as NC). The data in C and D are mean ± S.E.M. with six chickens per time point. The data in the other figures are mean ± S.E.M. with four cultures per time point or per group. For **A**–**G**, one-sample *t* test was used to assess the change from each data point to the control (50% in A and B, or GM in C and D); For **H**, **I** and **K**, one-way ANOVA with a Bonferroni post-hoc tests was used to analyze the data. * *p* < 0.05; ** *p* < 0.01; *** *p* < 0.001.

*Myomaker* up-regulates its expression during myoblast differentiation. This expression pattern was similar to those of *MYOG* and *MYOD* (Figure 5A,B), which encode important muscle-specific transcription factors [18,19]. Additionally, during chicken embryonic skeletal muscle development, *MYOG* expression was transiently up-regulated from E14-E16, similar to *Myomaker* (Figure 5C). However, the expression pattern of *MYOD* differed from those of *MYOG* and *Myomaker* (Figure 5D), suggesting that this gene has a different role in muscle development. Previous study of *MYOD* and *MYOG* demonstrated that these two transcriptional factors regulate the expression of skeletal muscle gene by binding to the E-box motifs located in gene promoters [20]. Therefore, we tested whether these two transcriptional factors can regulate *Myomaker* transcription. Established myotubes were transfected with siRNA designed specifically for *MYOG* and *MYOD* (Figure 5E,F). Knockdown of either of these two genes significantly reduced *Myomaker* mRNA expression, whereas overexpression of either *MYOG* or *MYOD* increased *Myomaker* mRNA expression (Figure 5E–G). These results indicate that MYOG and MYOD are able to regulate *Myomaker* expression directly or indirectly.

To investigate whether MYOG and MYOD can regulate *Myomaker* expression directly, we co-transfected the myoblasts with overexpression plasmids and luciferase reporter plasmids. The results showed that the overexpression of either *MYOG* or *MYOD* could significantly increase 0.6 kb promoter activity but that the E-box 1 mutation could eliminate this increase (Figure 5H), suggesting that MYOG and MYOD promote gene transcription by directly binding to the E-box 1 of the *Myomaker* promoter. Additionally, we also tested the luciferase activity of the two longer promoters in *MYOG*- or *MYOD*-overexpressing cells. No significant increases in the relative luciferase activity of these two longer promoters were observed (Figure 5I).

Next, ChIP-qPCR assays indicated that both MYOD and MYOG could bind to the promoter region (Region 1, R1), which contains E-box 1 and E-box 2 (Figure 5J,K). MYOD binds to the promoter abundantly on early differentiation day 1 (DM1) and late differentiation day 4 (DM4), whereas MYOG binding increased gradually from DM1 to DM4 (Figure 5K). The above results showed that MYOG and MYOD could directly bind to the *Myomaker* promoter, particularly to E-box 1, and induce *Myomaker* transcription during myoblast differentiation.

### 2.6. miR-140-3p Binds Directly to the 3ʹ UTR of Myomaker and Inhibits Myomaker Expression in Vitro

**Figure 6:**
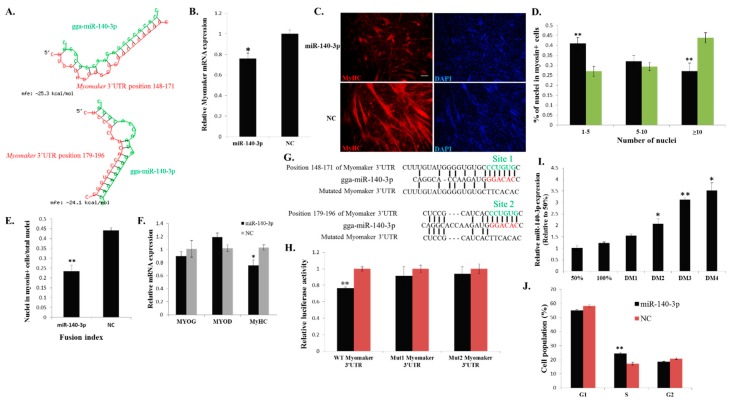
miR-140-3p directly binds to the 3ʹ UTR of *Myomaker* and inhibits *Myomaker* expression and myoblast fusion. (**A**) Schematic representation of the duplexes of miR-140-3p and the *Myomaker* 3ʹ UTR target region; (**B**) *Myomaker* mRNA expression after miR-140-3p mimic or NC duplexes transfection into DM2 myoblasts; (**C**) MyHC immunostaining of primary myoblasts transduced with miR-140-3p mimic or NC duplexes and differentiated for 48 h. Bar, 100 µm; (**D**) The distribution of myonuclear content in the cells with transfection of miR-140-3p mimic or NC duplexes; (**E**) The fusion index of the cells transfected with miR-140-3p mimic or NC duplexes; (**F**) The mRNA expression of *MYOG*, *MYOD* and *MyHC* after transfection of miR-140-3p mimic or NC duplexes in DM2 myoblasts; (**G**) Schematic representation of the predicted binding sites (green) and mutated sites of miR-140-3p in the 3ʹ UTR of *Myomaker*; (**H**) *Myomaker* 3ʹ UTR wild-type or mutant luciferase reporters were transfected into DF-1 cells overexpressing miR-140-3p mimic or NC duplexes. Luciferase activity was determined at 36 h after transfection; (**I**) miR-140-3p expression during myoblast differentiation; (**J**) Cell cycle analysis of myoblasts at 36 h after miR-140-3p mimic or NC duplexes transfection. The results are shown as the mean ±S.E.M. of at least three cultures (*n* = 3 cultures in **D**, **E** and **J**; *n* = 4 cultures in **B**, **F**, **H** and **I**). One-sample *t* test was used to assess the change from each data point to the control (50% in **I**). * *p* < 0.05; ** *p* < 0.01.

miRNAs can regulate gene expression by binding to the 3ʹ UTR of their target mRNAs [21]. To investigate whether any miRNAs are involved in regulating *Myomaker* during myoblast fusion, we used RNAhybrid [22] to identify potential miRNAs that can bind to the 3ʹ UTR of *Myomaker* mRNA. Among these miRNAs (Supplementary File 1), we found that miR-140-3p, which has two potential binding sites in *Myomaker* mRNA 3ʹ UTR (Figure 6A), can significantly inhibit *Myomaker* mRNA expression in primary myoblast (Figure 6B). Additionally, miR-140-3p overexpression during myoblast fusion inhibited cell fusion and led to smaller myotubes with less nuclei than the cells of the control (Figure 6C,D). The fusion index of miR-140-3p transfected cells significantly decreased (Figure 6E). Additionally, miR-140-3p overexpression did not alter the expression of *MYOG* and *MYOD* (Figure 6F). However, *MyHC* expression significantly decreased (Figure 6F), suggesting that *Myomaker* is not the only target of miR-140-3p that regulated myoblast fusion and differentiation, and that there is another target gene for miR-140-3p that can regulate *MyHC* expression and myoblast fusion.

The 3ʹ UTR of *Myomaker* mRNA has two predicted target sites for miR-140-3p (Figure 6G). Co-transfection of miR-140-3p with a *Myomaker* 3ʹ UTR dual-luciferase construct repressed luciferase activity significantly, and the mutation of each of the two target sites in the 3ʹ UTR relieved this repression (Figure 6H), indicating that miR-140-3p can directly bind to either of these two target sites in the *Myomaker* 3ʹ UTR. To our surprise, miR-140-3p expression gradually increased during the differentiation process (Figure 6I). This expression pattern was similar to those of *Myomaker*, *MYOG* and *MYOD*, suggesting that miR-140-3p may have another important function during myoblast differentiation. Because cell cycle arrest is important for myoblast differentiation, we next analysed whether miR-140-3p overexpression could regulate the cell cycle of myoblasts *in vitro*. Cell cycle analysis revealed that miR-140-3p transfected cells showed a lower percentage of G1 and G2 phase entries and a significantly higher percentage of S phase entry than cells transfected with control (Figure 6J and Supplementary File 2), demonstrating that miR-140-3p can regulate myoblast cell cycle progression *in vitro*. Altogether, in an *in vitro* system, miR-140-3p can inhibit *Myomaker* expression by binding directly to the 3ʹ UTR of *Myomaker* mRNA and can regulate myoblast cell cycle progression and differentiation by other target genes.

## 3. Discussion

In mice and zebrafish, Myomaker is a muscle-specific transmembrane protein with important roles in promoting myoblast fusion [11,13]. However, its roles in avians have not yet been elucidated. In this study, we first confirmed the important roles of *Myomaker* in chicken myoblast fusion and identified some of the regulatory mechanisms of its expression during myoblast fusion. Importantly, this study is the first to demonstrate that miR-140-3p can target inhibit *Myomaker* expression during myoblast differentiation. These findings not only provide evidence for the function and regulation of *Myomaker* during chicken myoblast fusion but also provide insight regarding the regulators and biological roles of *Myomaker*, which is essential for muscle formation and regeneration.

Our previous work demonstrated that E10-E16 is an important period of chicken muscle fibre formation [23]. In this study, we found that *Myomaker* mRNA expression is up-regulated during this period. The up-regulated expression of *Myomaker* may contribute to the promotion of myoblast fusion and muscle fibre formation. Studies show that the phenotype of sex-linked dwarf chicken is a result from a mutation of the *GHR* gene [24], and the mutation can lead to a decrease in muscle fibers number and fiber diameter [18]. To understand whether *Myomaker* is involve in the regulatory network of the *GHR* mutation induced muscle development difference, we tested its expression between the muscle of E14 dwarf and normal chickens. The results of reduced *Myomaker* mRNA expression in dwarf chickens suggesting that *Myomaker* may communicate with *GHR* gene by direct or indirect signalling pathway. Additionally, lower *Myomaker* expression in dwarf chickens may also result in the decrease of myoblast fusion during chicken muscle development. Therefore, *Myomaker* expression is important for chicken skeletal muscle development, and the *Myomaker* gene can be considered a candidate gene for molecular breeding in broilers.

Myomaker is a transmembrane protein. Previous studies have demonstrated that some transmembrane proteins can regulate myoblast fusion by influencing cell migration [6], recognition and adhesion [5,9,10], which are important processes during myoblast fusion [3]. Therefore, we examined whether Myomaker could regulate myoblast migration (Supplementary File 3). Results from Classic scratch and Transwell migration assays indicated that *Myomaker* had no significant effect on cell migration. Additionally, in *Myomaker* knockout mice, myoblasts are present in embryonic limbs [11], suggesting that *Myomaker* null myoblasts can migrate from somites to the limbs. Therefore, the above results indicate that Myomaker may be not a regulator of myoblast migration. Recently, a cell surface protein BAI3 was found to interact with ELMO and promote myoblast fusion by the ELMO-Dock1-Rac1 pathway in chick embryos [21]. This BAI3-ELMO-Dock1-Rac1 pathway is able to regulate the downstream actin cytoskeleton network, which plays an essential role during myoblast fusion [3]. As the actin cytoskeleton is also critical for Myomaker-induced myoblast fusion [11], it is possible that Myomaker can crosstalk with the pathway of BAI3-ELMO-Dock1-Rac1 to regulate myoblast fusion [21]. However, no related investigations have been performed to illustrate this interaction. The precise mechanism by which Myomaker regulates myoblast fusion still requires further exploration.

Although the function of Myomaker in myoblast fusion has been clearly demonstrated, the regulatory mechanism of *Myomaker* expression remains unknown. Myogenic regulatory factors (MRFs), which include MYOD, MYF5, MRF4 and MYOG, are able to activate many downstream genes to initiate muscle cell differentiation [14,15]. However, the specific roles of these factors are different. *MYF5* and *MYOD* act as myogenic determination genes, whereas *MYOG* is essential for the terminal differentiation of committed myoblasts [15,16,17]. *MRF4* seems to have both of these roles [25]. In this study, we tested the function of *MYOG* and *MYOD*, which are transcriptional factors that play essential roles in muscle-specific gene transcription [20,26], in the regulation of *Myomaker* transcription. Our results confirmed that both MYOG and MYOD can directly bind and activate *Myomaker* expression. However, in the luciferase assay, the luciferase activity of the 0.6-kb promoter only increased about 1.4 fold when overexpressing *MYOD* and *MYOG*. This may be due to the impact of endogenous MYOD and MYOG levels. As the deletion of E-box 1 can lower the promoter activity of pGL3-Myomaker 0.6kb (Figure 3B), its deletion can also reduce the promoter activity of pGL3-Myomaker 0.6kb when only pcDNA3.1-EGFP was transfected (Figure 4H). Moreover, other distal elements outside of the 2-kb promoter may be needed for a cooperative activation effect of the promoter, because many distal enhancers have been found to be involved in the activation of gene transcription.

Recently, Millay *et al.* found that two E-boxes located proximal to the *Myomaker* TSS were essential to *Myomaker* transcription in mice [12]. By analysing available ChIP-sequence data sets from the ENCODE Project, these authors revealed the significant binding of both MYOG and MYOD at these two E-boxes during C2C12 differentiation. Therefore, MYOG and MYOD can regulate *Myomaker* transcription directly in both mice and chicken.

A previous study showed that MYOD and MYOG play different roles in the regulation of a similar target genes set [20]. MYOD can initiate early gene expression and regional histone modification, whereas MYOG enhances the MYOD-initiated expression of a subset of genes. In our study, we found that MYOD was bound abundantly to the *Myomaker* promoter during both early and late differentiation. MYOG was bound less during early differentiation but bound more during late differentiation. Therefore, during early chicken myoblast differentiation, MYOD may first bind to the promoter of *Myomaker* and initiate *Myomaker* expression and regional histone modification. During late differentiation, MYOG binds to the promoter more efficiently with MYOD and then enhances the expression of *Myomaker*.

Many miRNAs have been found to be involved in skeletal muscle differentiation [27,28,29]. Here, we found that the miRNA miR-140-3p is another candidate involved in myoblast fusion *in vitro*. miR-140-3p can play roles in chondrogenic differentiation [30], testis differentiation [31] and spinal chordoma prognosis [32]. However, few studies have examined miR-140-3p involvement in muscle development. A previous study showed that miR-140-3p was immediately down-regulated in skeletal muscle within one hour of refeeding after fasting for one week [33], and target prediction and expressional analyses suggested that miR-140-3p might bind and inhibit the expression of the *myostatin* gene, which is a well-known negative regulator of muscle growth [34,35,36]. Another study in human airway smooth muscle cells showed that miR-140-3p attenuates the activation of NF-κB and p38 MAPK by indirect mechanisms [37]. Both NF-κB and p38 MAPK signalling are involved in skeletal muscle differentiation [38,39,40]; however, their specific roles are quite different. p38 MAPK signalling is a positive regulator in muscle development [38,39], whereas the data from knockout mice support that the NF-κB pathway functions as an inhibitor of myogenesis [41]. Therefore, the function of miR-140-3p in muscle remains to be explored.

In our results, miR-140-3p overexpression inhibited *Myomaker* and *MyHC* expression. The *Myomaker* gene is a direct target of miR-140-3p. However, the mechanism of miR-140-3p inhibited *MyHC* expression remains unclear. It is possible that there are other target genes of miR-140-3p that can regulate *MyHC* expression, because *Myomaker* loss-of-function has no impact on *MyHC* expression. Additionally, miR-140-3p expression during myoblast differentiation is consistent with *Myomaker*, suggesting that this miRNA may have another function in this process. Cell cycle arrest is an important event for myoblast differentiation, and our results showed that miR-140-3p promotes myoblast proliferation, suggesting the negative role of miR-140-3p in myoblast differentiation. However, the roles and expression pattern of miR-140-3p are similar to those of miR-133a, which is an important muscle-specific miRNA during muscle development [42,43,44]. miR-133a has sharply increased expression during muscle differentiation and functions not only in the inhibition of muscle differentiation but also in the promotion of myoblast proliferation [42,43]. Therefore, miR-140-3p may be a positive regulator during muscle development similar to miR-133a. However, the regulatory mechanism of miR-140-3p during myoblast differentiation remains unclear, and its regulatory role in *Myomaker* is limited to the *in vitro* system. The specific function and mechanism of miR-140-3p in myoblast differentiation and proliferation remains to be further explored.

## 4. Experimental Section

### 4.1. Animals and Cells

The chicken embryos used in this study were as previously described [23]. The primary myoblasts of chicken were isolated from the leg muscles of E10 chicks and maintained in cell culture using growth medium as previously characterized [23]. Myoblast differentiation was induced by culture in differentiation medium, which consisting of DMEM medium (Gibco, Grand Island, NY, USA), 2% horse serum (Hyclone, Logan, UT, USA) and 0.2% penicillin/streptomycin (Invitrogen, Carlsbad, CA, USA). DF-1 cells were cultured in DMEM with 10% fetal bovine serum and 0.2% penicillin/streptomycin.

### 4.2. cDNA Synthesis and Quantitative Real-Time PCR (qPCR)

Total RNA from tissues or cells was extracted using RNAiso reagent (Takara, Otsu, Japan). cDNA synthesis for mRNA was using PrimeScript^TM^ RT reagent Kit (Perfect Real Time) (Takara, Otsu, Japan). qPCR program was performed in a Bio-rad CFX96 system (Bio-Rad, Hercules, CA, USA) using KAPA SYBR^®^ FAST qPCR Kit (KAPA Biosystems, Woburn, MA, USA). The relative expression level was calculated using the method as described before [45]. qPCR primers sequences for all genes are listed in Supplementary File 4.

### 4.3. The 5ʹ and 3ʹ Rapid Amplification of cDNA Ends (RACE)

For 5ʹ RACE, total RNA isolated from chicken embryo skeletal muscle was reversely transcribed using 5ʹ RACE RT-adapter primer and PrimeScript^TM^ II Reverse Transcriptase (Takara, Otsu, Japan). The obtained first-strand cDNA was subsequently digested by RNase H (Takara, Otsu, Japan) and tailed with terminal deoxynucleotidyl transferase (Beyotime, Shanghai, China) and dCTP. Two rounds of PCR were performed to amplify reverse transcribed products. First round with 5ʹ-RACE outer primer corresponding to RT-adapter and a *Myomaker* specific outer primer, and a second round PCR with 5ʹ-RACE inner primer and a nested *Myomaker* specific inner primer. For 3ʹ RACE, the synthesis of first-strand cDNA was carried out using the Oligo(dT)-anchor primer and PrimeScript^TM^ II Reverse Transcriptase (Takara, Otsu, Japan). The following PCR amplification was performed using Myomaker specific outer primer and the 3ʹ-adaptor outer primer, and further nested with *Myomaker* specific inner primer and 3ʹ-adaptor inner primer. The above PCR products were then gel-purified, ligated into pGEM-T Easy vector (Promega, Madison, WI, USA) and sequenced. All of the primers used in RACE were summarized in Supplementary File 4.

### 4.4. Immunofluorescence

Primary myoblasts seeded in 24-well plates were cultured to 100% confluence and then transfected. Forty-eight hours after transfection with miRNA, siRNA or overexpression vector, the cells were fixed and stained for MyHC and DAPI (Beyotime, Shanghai, China) as previously described [23]. Images were captured using Nikon Eclipse Ti-U fluorescent microscope.

### 4.5. ChIP Assays

ChIP assays were carried out using ChIP Assay Kit (Beyotime, Shanghai, China) according to the manufacturer’s instructions. One μg of MYOD (BD Biosciences, San Jose, CA, USA), MYOG (Biorbyt, Cambridge, UK) or control IgG antibody were used in immunoprecipitation. ChIP products were subjected to quantitative PCR using a KAPA SYBR^®^ FAST qPCR Kit (KAPA Biosystems, Woburn, MA, USA). The primer sequences for ChIP-qPCR analysis are listed in Supplementary File 4.

### 4.6. Transfections

Transfection was carried out using Lipofectamine 3000 reagent (Invitrogen, Carlsbad, CA, USA). Cells were transfected with 50 nM miRNA mimics (RiboBio, Guangzhou, China) or 100 nM siRNA (GenePharma, Suzhou, China). Lipofectamine 3000 and nucleic acids were diluted in OPTI-MEM I Reduced Serum Medium (Gibco, Grand Island, NY, USA). The procedure of transfection was performed according to the manufacturer’s direction.

### 4.7. Plasmid Construction

#### 4.7.1. pcDNA-3.1 Gene Overexpression Vectors

The coding sequences of chicken *Myomaker*, *MYOG* and *MYOD* were amplified using gene-specific clone primers (Supplementary File 4) and then cloned into the vector of pcDNA-3.1 (Invitrogen, Carlsbad, CA, USA) or pcDNA-3.1-EGFP.

#### 4.7.2. pmirGLO Dual-Luciferase Reporters

*Myomaker* 3ʹ UTRs were amplified from chicken embryonic leg muscle cDNA and ligated into the pmirGLO vector (Promega, Madison, WI, USA). The mutant Myomaker-3ʹ UTR reporters were generated by changing the miR-140-3p binding site from CCTGTG to TTCACA, and mutagenesis was carried out by PCR amplification and *Dpn*I digestion to remove the parental DNA.

#### 4.7.3. *Myomaker* Promoter Reporter Plasmid

A 2-kb fragment of the *Myomaker* promoter was isolated by PCR using the primers listed in Supplementary File 4. After the PCR product was digested with *Kpn*I and *Sma*I, the insertion was ligated into the pGL3-basic vector (Promega, Madison, WI, USA) to create the expression vector pGL3-Myomaker-2K. After pGL3-Myomaker-2K was sequenced, this construct was used as a template, and pGL3-Myomaker-1.3K or pGL3-Myomaker-0.6K was isolated by PCR. Site-directed mutagenesis of E-box 1 and E-box 2 were carried out by PCR amplification and *Dpn*I digestion to remove the parental DNA.

### 4.8. Target Prediction

RNAhybrid algorithm (http://bibiserv2.cebitec.uni-bielefeld.de/rnahybrid) was used to predict miRNAs potential target sites for *Myomaker* mRNA 3ʹ UTR. The default settings was used to run the algorithm with the extra constraints of perfect base pairing in the seed sequence of miRNA (nucleotides 2 to 7) and with the binding minimum free energy (mfe) lower than −20 kcal/mol.

### 4.9. Dual Luciferase Reporter Assay

For Myomaker promoter assays, myoblasts were transfected with reporter plasmid or co-transfected with overexpression vectors for MYOG and MYOD, and the TK-Renilla reporter (Promega, Madison, WI, USA) was co-transfected to each sample as an internal control. For miRNA target validation assays, wild-type or mutant Myomaker 3ʹ UTR dual-luciferase reporter (100 ng) and miR-140-3p mimic or NC duplexes (50 nM) were co-transfected into DF-1 cells using the Lipofectamine 3000 reagent (Invitrogen, Carlsbad, CA, USA) in 96-well plates. After the cells were transfected for 36 h, luciferase activities were measured according to the manufacturer’s instructions (Dual-luciferase reporter assay system; Promega, Madison, WI, USA). Synergy 2 Multi-mode Microplate Reader (Biotek, Winooski, VT, USA) was used to quantify the luminescent signal and analysed using Gene5 software (Biotek, Winooski, VT, USA).

### 4.10. Cell-Cycle Analysis

After 36 h transfection, cells culture in growth medium were collected, fixed, permeabilized and stained with propidium iodide (Sigma, St. Louis, MO, USA) containing 10 µg/mL RNase A (Takara, Otsu, Japan) for flow cytometric cell cycle analysis using a BD Accuri C6 flow cytometer (BD Biosciences, San Jose, CA, USA). Data were analysed using FlowJo 7.6 software (Verity Software House, Tosham, ME, USA).

### 4.11. Statistical Analysis

Unless otherwise stated, all results are showed as mean ± S.E.M. At least three independent experiments were performed for each treatment. Statistical significance between groups was analyzed by one-sample *t* tests or ANOVA.

### 4.12. Ethics Standards

All animal experiments were carried out with the permission of the Animal Care Committee of South China Agricultural University (approval number: SCAU#0014). The experiment was performed in accordance with the regulations and guidelines established by this committee.

## 5. Conclusions

In conclusion, our study confirms the important role of Myomaker in chicken myoblast fusion, and finds that MYOD and MYOG directly bind to the conserved E-box 1 located proximal to the *Myomaker* transcription start site and induces *Myomaker* mRNA transcription. Moreover, we found for the first time that miR-140-3p can inhibit *Myomaker* expression and myoblast fusion, at least in part, by binding to the 3ʹ UTR of *Myomaker*. miR-140-3p can also regulate the cell cycle of myoblasts. Therefore, these results not only demonstrate that Myomaker regulates avian myoblast fusion, but also that three regulators, MYOD, MYOG and miR-140-3p, can influence *Myomaker* expression during myoblast differentiation.

## Supplementary Materials

Supplementary materials can be found at http://www.mdpi.com/1422-0067/16/11/25946/s1.
