# Supplementary material for: Myomaker, Regulated by MYOD, MYOG and miR-140-3p, Promotes Chicken Myoblast Fusion
Source: Int J Mol Sci. 2015 Nov 2;16(11):26186–201. doi: 10.3390/ijms161125946 (PMC4661805; doi:10.3390/ijms161125946)
Supplement: Supplementary file 1 [file ijms-16-25946-s001.pdf]

International Journal of Molecular Sciences

MDPI

Supplementary Information

Supplementary File 1.

target: myomaker
length: 387
miRNA : gga-miR-133b
length: 21

mfe: -28.5 kcal/mol

```
position    230
target 5'   U        CCC       A           A 3'
                GCUGG       AGG GGACCAG
                CGACC       UCC CCUGGUU
miRNA    3' AU        AACU                 5'
```
----

target: myomaker
length: 387
miRNA : gga-miR-1659
length: 23

mfe: -25.1 kcal/mol

```
position    139
target 5' U    A    AG           AUGGGG G           U 3'
               GG GA    CCUUUGU          U UGCCCUG
               CC CU    GGAAGUA          A ACGGGAU
miRNA    3' U        GA                   G             5'
```
----

target: myomaker
length: 387
miRNA : gga-miR-140-3p
length: 21

mfe: -25.3 kcal/mol

```
position    148
target 5' C UU    AU    GG G             C 3'
               U    GU    GG   U UGCCCUGUG
               A    CA    CC   A AUGGGACAC
miRNA    3' C GG          A    G             C 5'
```
----

target: myomaker
length: 387

miRNA : gga-miR-140-3p
length: 21

mfe: -24.1 kcal/mol

position 179
target 5' C   CA   C 3'
          UCCG   UC ACCCUGUG
          AGGC   AG UGGGACAC
miRNA 3' C   ACCA A   C 5'
----
target: myomaker
length: 387
miRNA : gga-miR-6543-3p
length: 18

mfe: -21.1 kcal/mol

position 232
target 5' C   C   A 3'
           UGGCC   AGGAGG
           ACUGG   UCCUCC
miRNA 3' UC   ACUU   G 5'
----
target: myomaker
length: 387
miRNA : gga-miR-1580
length: 21

mfe: -23.2 kcal/mol

position 113
target 5' U   UUUCACUU   AAAUUCCCCUG   U 3'
          GCGC   UGCU   GAGAAGCC
          UGCG   AUGG   CUCUUCGG
miRNA 3' U   UCC   C 5'
----
target: myomaker
length: 387
miRNA : gga-miR-6650-5p
length: 24

mfe: -29.1 kcal/mol

position 196
target 5' C   UUG   CACUUCAC CCCCCUGUCCCC   C 3'
          GCCC   GC   CU   UCUGCUGGC
          CGGG   CG   GA   AGACGACCG

miRNA 3' A UCAA A A 5'
----
target: myomaker
length: 387
miRNA : gga-miR-1768
length: 22

mfe: -25.8 kcal/mol

position 6
target 5' C A G C 3'
CA CACGG U CUCUGCUC
GU GUGCU A GAGACGAG
miRNA 3' A C GUU G 5'
----

target: myomaker
length: 387
miRNA : gga-miR-1608
length: 22

mfe: -28.9 kcal/mol

position 174
target 5' G CUCCGCAUCACCCU U CCUU UUCACCUCCCCC C 3'
AGAG G GCGC GGCCAC UGUCCC
UCUC C CGCG UCGGUG ACAGGG
miRNA 3' U 5'
----
target: myomaker
length: 387
miRNA : gga-miR-1789
length: 22

mfe: -25.6 kcal/mol

position 277
target 5' A UU A G 3'
UC UCUGU GU ACACCCA
AG AGGCG CA UGUGGGU
miRNA 3' GUA U UU 5'
----
target: myomaker
length: 387
miRNA : gga-miR-20a-5p
length: 23

mfe: -20.9 kcal/mol

```
position    107
target 5'   A     GA         UU               C 3'
                GCU   UGCGCU       CACUUUG
                UGG   ACGUGA       GUGAAAU
miRNA   3' GA                 UAUUC              5'
```
----
target: myomaker
length: 387
miRNA : gga-miR-1684a-5p
length: 22

mfe: -23.5 kcal/mol

```
position    232
target 5'    C    GCCCAG        C             U 3'
                 UG          GAGGA    CAGAGCU
                 AC          CUCCU    GUCUCGA
miRNA   3' CAU    AUA             UC              5'
```
----
target: myomaker
length: 387
miRNA : gga-miR-1722-3p
length: 22

mfe: -28.2 kcal/mol

```
position    1
target 5'            CCAACAC             C 3'
                 GCCCC          GGUGCUCUG
                 CGGGG          CUACGAGAU
miRNA   3' GAA        AACCA                     5'
```
----
target: myomaker
length: 388
miRNA : gga-miR-1555-5p
length: 20

mfe: -23.4 kcal/mol

```
position    58
target 5'   C        CUUUUCUUCUGUG     U             C 3'
                CAGGG                    GGA GGUUUGCU
                GUCCC                    CUU CCAAACGA
miRNA   3' CC        A                     C                5'
```
----
target: myomaker
length: 388
miRNA : gga-miR-1582

length: 20

mfe: -25.7 kcal/mol

```
position    71
target 5' U         GGA       UUG            C 3'
               CUGUG     UGGU     CUCUUUC
               GACAC     ACCG     GAGAAAG
miRNA   3'           AAG       A                  5'
```

----

target: myomaker
length: 388
miRNA : gga-miR-133b
length: 21

mfe: -28.5 kcal/mol

```
position    230
target 5'    U         CCC        A            A 3'
                GCUGG       AGG GGACCAG
                CGACC       UCC CCUGGUU
miRNA   3' AU         AACU                     5'
```

----

target: myomaker
length: 388
miRNA : gga-miR-133c-3p
length: 22

mfe: -28.5 kcal/mol

```
position    230
target 5'     U         CCC        A            A 3'
                 GCUGG       AGG GGACCAG
                 CGACC       UCC CCUGGUU
miRNA   3' CGU         AACU                     5'
```

----

target: myomaker
length: 388
miRNA : gga-miR-1732
length: 21

mfe: -23.8 kcal/mol

```
position    1
target 5'            CC         A            C 3'
```

```
                GCCC      CA CACGGUG
                CGGG      GU GUGCCAC
miRNA   3' GU     CUGUC   C          5'
```

----
target: myomaker
length: 388
miRNA : gga-miR-6665-5p
length: 22

mfe: -22.3 kcal/mol

```
position   233
target 5' U    C       GGACCAGAG        C 3'
              GGC   CAGGA          CUUCAGGC
              CCG   GUUUU          GAAGUCCG
miRNA   3' C    UA      AG                U 5'
```

----
target: myomaker
length: 388
miRNA : gga-miR-1594
length: 21

mfe: -29.6 kcal/mol

```
position   20
target 5' U        CGGG     U G       G 3'
              GCUCCA      AUCU U CCCACCA
              UGGGGU      UGGG A GGGUGGU
miRNA   3' U                 U G          5'
```

----
target: myomaker
length: 388
miRNA : gga-miR-6675-3p
length: 22

mfe: -26.0 kcal/mol

```
position   225
target 5' C      U   UG CCCAG   G A       C 3'
              CCUC GC   G      GA G CCAGAG
              GGAG UG   C      CU C GGUCUC
miRNA   3'           UG A       G A       U 5'
```

----
target: myomaker
length: 388

miRNA : gga-miR-6675-3p
length: 22

mfe: -22.2 kcal/mol

position 146
```
target 5' G      UGUAUG G    UGCCC    G          G 3'
           CCUU        G GUG      UGU CCAGAGA
           GGAG        U CAC      GCA GGUCUCU
miRNA  3'       UG       G    U                 5'
```

----
target: myomaker
length: 388
miRNA : gga-miR-1744-5p
length: 22

mfe: -20.5 kcal/mol

position 230
```
target 5' U    GGCCCAG    GAC          U 3'
           GCU        GAG       CAGAGC
           CGA        CUC       GUCUCG
miRNA  3' U    AAA        AAGUU        U 5'
```

----
target: myomaker
length: 388
miRNA : gga-miR-7482-5p
length: 25

mfe: -31.1 kcal/mol

position 227
```
target 5' C    G     CCCAG    GACCA    UUC          U 3'
           UCU CUGG      GAG      GAGC    AGGCCCAG
           AGA GACC      CUC      CUUG    UUCGGGUC
miRNA  3'          A         A                    C 5'
```

----
target: myomaker
length: 388
miRNA : gga-miR-7482-5p
length: 25

mfe: -26.6 kcal/mol

position 31

target 5' A   CCCACC   UCCAU   C   G 3'
            UCUUUG   AGU   ACG GCCCAGG
            AGAGAC   UCA   UGU CGGGUCC
miRNA 3'   CAC   CU   U   5'

----

target: myomaker
length: 388
miRNA : gga-miR-6562-5p
length: 22

mfe: -25.3 kcal/mol

position 108
target 5' G UG U   U   A   UUGCUAAA   G 3'
            C   A GCGC UUC CU   UUCCCCU
            G   U CGUG AAG GA   AAGGGGA
miRNA 3' G UG   U   5'

----

target: myomaker
length: 388
miRNA : gga-miR-99a-3p
length: 21

mfe: -28.7 kcal/mol

position 233
target 5' U   ACCA   C 3'
            GGCCCA GGAGG   GAGCUU
            CUGGGU UCUUC   CUCGAA
miRNA 3' U   A   G   C 5'

----

target: myomaker
length: 388
miRNA : gga-miR-20b-5p
length: 23

mfe: -21.9 kcal/mol

position 107
target 5'   A   GA   UU   C 3'
              GCU   UGCGCU   CACUUUG
              UGG   ACGUGA   GUGAAAC
miRNA 3' GA   UACUC   5'

----
target: myomaker
length: 388
miRNA : gga-miR-7454-5p
length: 23

mfe: -23.1 kcal/mol

```
position    250
target 5'    A          GCCCA      GAGCA        U 3'
                GCUUCAG       GUUU       CACAGA
                UGAAGUC       CAGA       GUGUCU
miRNA   3' CG          AAA                      U 5'
```

----
target: myomaker
length: 388
miRNA : gga-miR-6637-3p
length: 23

mfe: -27.8 kcal/mol

```
position    234
target 5' G    CAG   GGAC    A   UUCA           U 3'
              GCC    GA     CAG GC      GGCCCAGU
              UGG    CU     GUC CG      UCGGGUCG
miRNA   3'              AAC         UG                5'
```

----
target: myomaker
length: 388
miRNA : gga-miR-1736-5p
length: 22

mfe: -27.6 kcal/mol

```
position    12
target 5' C       U     CUCCA       UCU         A 3'
              GGUGC CUG       CGGGA    UUGCCC
              UCGUG GAU       GUCCU    AACGGG
miRNA   3'               A           U           A 5'
```

----
target: myomaker
length: 388
miRNA : gga-miR-7439-3p
length: 23

mfe: -25.8 kcal/mol

```
position    113
target 5' U          UCACUUU       AAUUCCC             G 3'
               GCGCUU          GCUA           CUGGAGAA
               CGUGAG          UGAU           GACCUCUU
miRNA    3' U          UCCU                              5'
```

----

target: myomaker
length: 388
miRNA : gga-miR-757
length: 21

mfe: -27.7 kcal/mol

```
position    4
target 5'       C       ACAC GU            U 3'
                   CCCA      G    GCUCUGC
                   GGGU      C    CGAGACG
miRNA    3' CUUA       AGA    GU               5'
```

----

target: myomaker
length: 388
miRNA : gga-miR-214
length: 21

mfe: -24.7 kcal/mol

```
position    218
target 5'     C          CCC             G 3'
                 CCUGUC      UCUGCUG
                 GGACAG      GGACGAC
miRNA    3' GAC          ACAC            A 5'
```

----

target: myomaker
length: 388
miRNA : gga-miR-7472-5p
length: 22

mfe: -26.8 kcal/mol

```
position    245
target 5' A    AG            GGCCCAG     G          C 3'
               CC    AGCUUCA            UUU AGCACA
               GG    UCGAAGU            GAG UCGUGU
miRNA    3' A                  A              G          A 5'
```

----
target: myomaker
length: 388
miRNA : gga-miR-6672-3p
length: 20

mfe: -23.7 kcal/mol

position 145
target 5' A C GUA GG U 3'
G CUUU UGG UGUGCCC
C GAAG GUC ACACGGG
miRNA 3' A A AA U 5'

----
target: myomaker
length: 388
miRNA : gga-miR-7452-5p
length: 21

mfe: -30.3 kcal/mol

position 151
target 5' U GU G 3'
GUAUGG GGU GCCCUGU
CGUGCC UCG CGGGACA
miRNA 3' UAG GU 5'

----
target: myomaker
length: 388
miRNA : gga-miR-6625-5p
length: 22

mfe: -24.0 kcal/mol

position 196
target 5' C C GCCA ACC U 3'
GC CUUG CUUC UCCCCC
UG GAAC GAAG AGGGGG
miRNA 3' A GUCA U 5'

----
target: myomaker
length: 388
miRNA : gga-miR-1773-5p
length: 23

mfe: -29.9 kcal/mol

position 196
target 5' C CCUU A UCA U 3'
GC GG CC CU CCUCCCCC
CG CC GG GA GGAGGGGG
miRNA 3' UU UCU A U 5'

----
target: myomaker
length: 388
miRNA : gga-miR-1757
length: 21

mfe: -24.9 kcal/mol

position 211
target 5' C CCCC C 3'
ACCU CUG UCC CCUCUG
UGGA GAC AGG GGAGAC
miRNA 3' U U A C U 5'

----
target: myomaker
length: 388
miRNA : gga-miR-133a-3p
length: 22

mfe: -28.5 kcal/mol

position 230
target 5' U CCC A A 3'
GCUGG AGG GGACCAG
CGACC UCC CCUGGUU
miRNA 3' UGU AACU 5'

----
target: myomaker
length: 388
miRNA : gga-miR-1704
length: 21

mfe: -26.1 kcal/mol

position 217
target 5' C CCC G 3'
CCCUGUC UCUGCUG
GGGGUAG AGACGAC

miRNA 3' ACAC UU A 5'

----
target: myomaker
length: 388
miRNA : gga-miR-6558-5p
length: 24

mfe: -24.0 kcal/mol

position 85
target 5' U UCUUU A GCCCGAGCUGAU C U 3'
GC CCACC UCA GCG UUUCACUU
UG GGUGG AGU UGC AAAGUGAA
miRNA 3' UUC 5'

----
target: myomaker
length: 388
miRNA : gga-miR-17-5p
length: 24

mfe: -22.4 kcal/mol

position 107
target 5' A A UU C 3'
GCUG UGCGCU CACUUUG
UGAU ACGUGA GUGAAAC
miRNA 3' GG CAUUC 5'

----
target: myomaker
length: 388
miRNA : gga-miR-6596-3p
length: 22

mfe: -32.5 kcal/mol

position 154
target 5' A UG CUGUG AGA C 3'
UGGGG UGCC CC GAGCUCCG
GCCCU GCGG GG CUCGAGGC
miRNA 3' A CA 5'

----
target: myomaker
length: 388
miRNA : gga-miR-3540
length: 22

mfe: -26.0 kcal/mol

position 60
target 5' A UCUUCUGUGGGAUG GCUCU C 3'
GGGCUUU GUUU UUCCACCAU
UCCGGAA CAAG AAGGUGGUA
miRNA 3' CG 5'

----
target: myomaker
length: 388
miRNA : gga-miR-6645-5p
length: 22

mfe: -31.9 kcal/mol

position 185
target 5' A CUGUGCGCCCUUG U C 3'
UCACC GCCACU C ACCUCCC
AGUGG UGGUGA G UGGAGGG
miRNA 3' C A U 5'

----
target: myomaker
length: 388
miRNA : gga-miR-6647-5p
length: 23

mfe: -28.2 kcal/mol

position 230
target 5' U C GGA A U 3'
GCUG G C CA GG CCAGAGC
CGAC C G GU CC GGUCUCG
miRNA 3' U G A U A C 5'

----
length: 388
miRNA : gga-miR-3524b-3p
length: 21

mfe: -22.5 kcal/mol

position 42
target 5' C UU ACGCG G CUUUUC G 3'
CAG CCAU CCCA GG UUCUGUG
GUC GGUA GGGU UU AAGACAC

```
miRNA   3'                    G                 5'
```

----
target: myomaker
length: 388
miRNA : gga-miR-1790
length: 20

mfe: -30.6 kcal/mol

```
position   161
target 5' G G    UG      AGAGAGCUCCGCA        U 3'
              U CCC   UGCC              UCACCC
              G GGG   GCGG              AGUGGG
miRNA   3' A G    UG      G                    C 5'
```

----
target: myomaker
length: 388
miRNA : gga-miR-1680-5p
length: 22

mfe: -28.6 kcal/mol

```
position   229
target 5'          C   CUG            C 3'
                     UG    GCCCAGGAGGA
                     AC    UGGGUCCUCCU
miRNA   3' CGUCUCA   A               U 5'
```

----
target: myomaker
length: 388
miRNA : gga-miR-7458-3p
length: 22

mfe: -26.9 kcal/mol

```
position   226
target 5' C          GCCCAGGAGGACC    GCUUCAGG       U 3'
              CUCUGCUG               AGA         CCCAGU
              GAGACGAC               UCU         GGGUCA
miRNA   3'                           ACUA            C 5'
```

----
target: myomaker
length: 388
miRNA : gga-miR-6669-5p
length: 24

mfe: -28.8 kcal/mol

```
position    160
target 5' U       CCUGU     AGAGA          C          C 3'
                GUGC        GCC        GCUC CG AUCACC
                UACG        CGG        CGAG GC UAGUGG
miRNA    3'          U          AA          G    A            5'
```

----
target: myomaker
length: 388
miRNA : gga-miR-1728-3p
length: 22

mfe: -23.1 kcal/mol

```
position    105
target 5'     C A        UGCGCUUUCAC        UAAAUUCCCCUGG            U 3'
                  G GCUGA                UUUGC                  AGAAGCCU
                  C CGACU                AGAUG                  UCUUCGGA
miRNA    3' CGA                                                      5'
```

Supplementary File 2.

A. The used in the main figures of full-gels are shown. All cropped gels have been run under the same experimental condition.

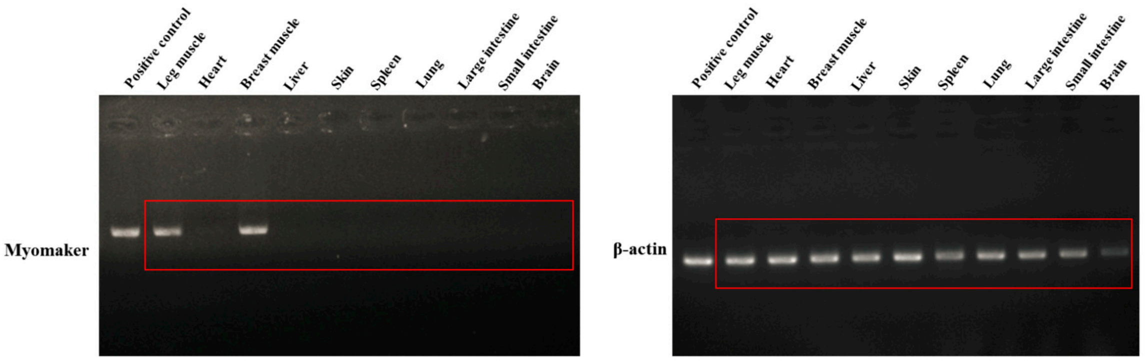

B. The histograms of cell cycle analyzed by flow cytometry.

miR-140-3p

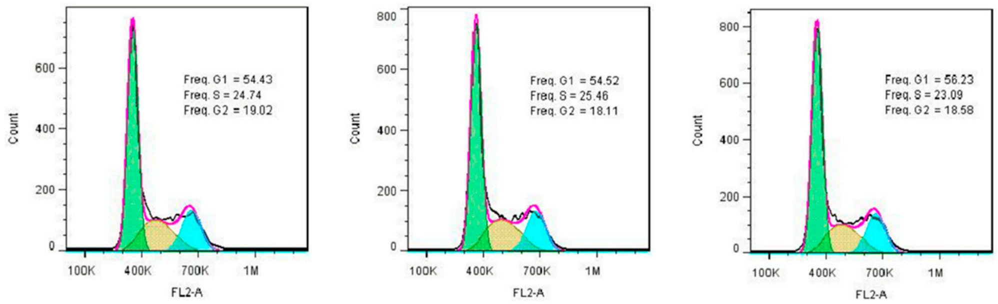

NC

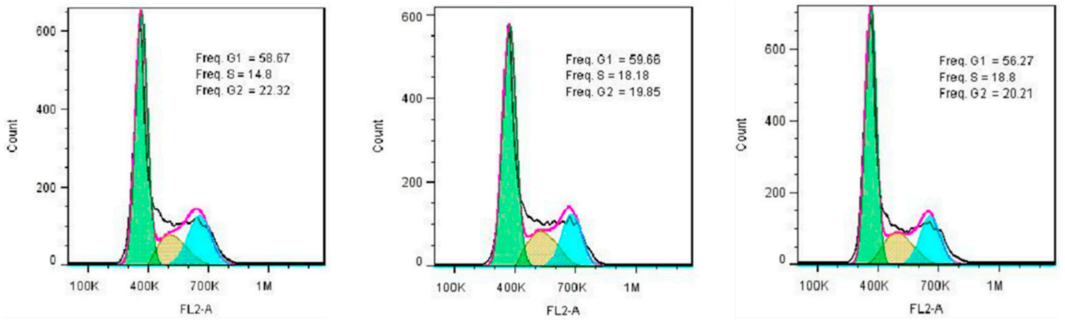

Supplementary File 3.

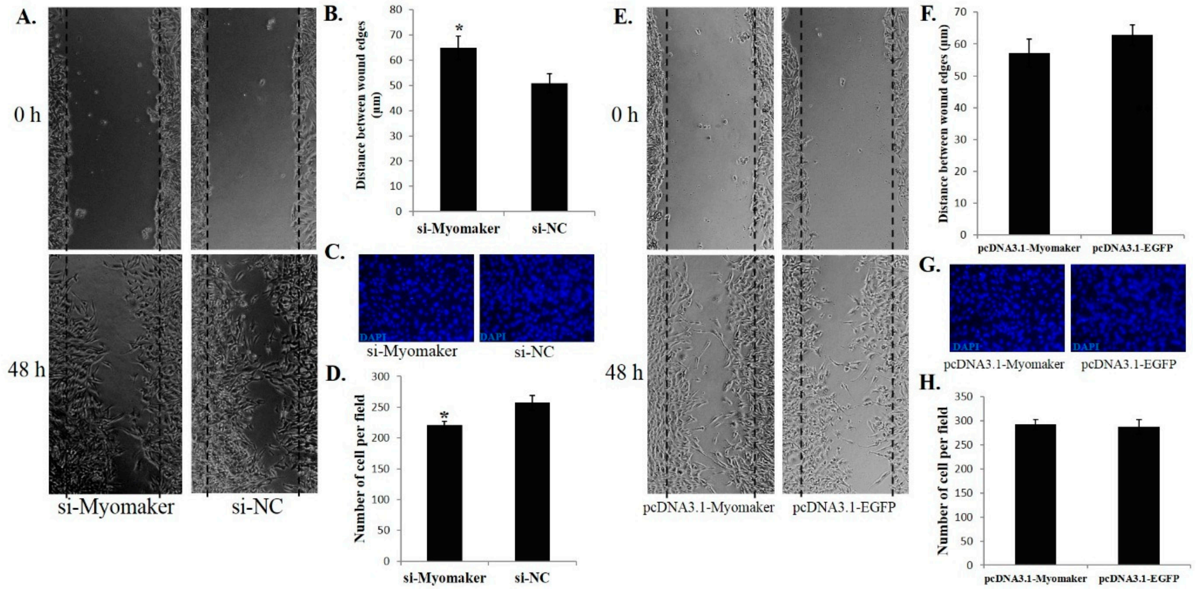

A. Representative classic scratch assay results for myoblasts transfected with si-Myomaker or si-NC. **B**. The statistical results of the distance between wound edges in the classic scratch assay for myoblasts transfected with si-Myomaker or si-NC. **C**. Transwell migration assay using myoblasts transfected with si-Myomaker or si-NC. **D**. The statistical results for the cell number in the Transwell migration assay for myoblasts transfected with si-Myomaker or si-NC. **E**. Representative classic scratch assay results for myoblasts transfected with pcDNA3.1-Myomaker or pcDNA3.1-EGFP. **F**. The statistical results of the distance between wound edges for the classic scratch assay for myoblasts transfected with pcDNA3.1-Myomaker or pcDNA3.1-EGFP. **G**. Transwell migration assay using myoblasts transfected with pcDNA3.1-Myomaker or pcDNA3.1-EGFP. **H**. The statistical results of the cell number for the Transwell migration assay for myoblasts transfected with pcDNA3.1-Myomaker or pcDNA3.1-EGFP.

Methods

Classic scratch assay: A ~400-μm scratch was made using a sterile pipette tip on a fully confluent cell monolayer 12 h after transfection with siRNA oligos or pcDNA3.1-Myomaker. Then the cells were washed and cultured in growth media. Images were taken using a phase contrast microscope (Nikon). The distance between wound edges were measured using NIS-Elements BR analysis software (Nikon).

Transwell migration assay: A total of 5×10^4 cells in 250 μL sera-free media were seeded in an upper chamber of a noncoated Transwell insert (24-well insert; pore size, 8 μm; BD Biosciences). Media supplemented with serum was used as the chemoattractant in the lower chamber. After 24-h

incubation, cells in the upper chamber were removed with a cotton swab and cells which migrated through the pores were fixed and stained with DAPI (Beyontime). Images of migrated cells were taken with a fluorescence microscope (Nikon) and the numbers of migrated cells were quantified with ImageJ (National Institutes of Health).

Supplementary File 4.

Oligonucleotides:
For sex determination the following primers were used for PCR:
CHD1 s: 5'-GTTACTGATTCGTCTACGAGA
as: 5'-ATTGAAATGATCCAGTGCTTG

For ChIP-qPCR analysis the following primers were used:
Myomaker promoter s: 5'-GTGCATCACAGCCAGCATG
as: 5'-GGTGGGCAGGAGGAGTTT
GAPDH s: 5'-ATGGCATCCAAGGAGTGA
as: 5'-GGGAGACAGAAGGGAACAG

For cloning the following primers were used for PCR:
Myomaker CDS cloning for pcDNA3.1: s: 5'-CCGCTCGAGGCCACCATGGGCTCGCTGGTGG
as: 5'-CGGGGCCCGCGTATGGAACTGGTGGG
Myomaker CDS cloning for pcDNA3.1-EGFP:
s: 5'-CCCAAGCTTGCCACCATGGGCTCGCTGGTGG
as: 5'-GGGGTACCGACACAGCAGCACAGCGTGGA
MYOG CDS cloning: s: 5'-CCGCTCGAGGCCACCATGGAGCTCTTTGAG
as: 5'-CGGGGCCCACTTGGAAACAGCCACATTG
MYOD CDS cloning: s: 5'-GGAATTCGCCACCATGGACTTACTGGGCCCCATGGAA
as: 5'-CCGCTCGAGGGCTGAACGGAGCAATT

Myomaker 3'UTR Fragment:
s: 5'-CCGCTCGAGTTGCCCACCAGTTCCAT
as: 5'-ACGCGTCGACTTGCACGTTTGCCAAGT
Myomaker 0.6kb promoter s: 5'-CGGGGTACCAGTGATCTGCTCCTATTCCTCC
as: 5'-TCCCCCGGGGCGATGCTGATGGTGGG
Myomaker 1.3kb promoter s: 5'-CGGGGTACCCCTGTGAAGGGATGCTCG
as: 5'-TCCCCCGGGCAAAGTGCTGAGGGTGGG
Myomaker 2.0kb promoter s: 5'-CGGGGTACCTAGCCCTGTGGAATGTGAAT
as: 5'-TCCCCCGGGCTGTTGGAGATGGAGGAGAA

For quantitative real time RT-PCR, the following primers were used:
Myomaker-qPCR: s: 5'-TGGGTGTCCCTGATGGC
as: 5'-CCCGATGGGTCCTGAGTAG
MYOD-qPCR: s: 5'-GCTACTACACGGAATCACCAAAT
as: 5'-CTGGGCTCCACTGTCACTCA
MYOG-qPCR: s: 5'-CGGAGGCTGAAGAAGGTGAA
as: 5'-CGGTCCTCTGCCTGGTCAT
MyHC-qPCR: s: 5'-CTCCTCACGCTTTGGTAA
as: 5'-TGATAGTCGTATGGGTTGGT
β-actin-qPCR: s: 5'-ATCTTTCTTGGGTATGGAGTC
as: 5'-GCCAGGGTACATTGTGG

For mutagenesis the following primers were used for PCR:
myomaker-3'UTR-mut1:
s: 5'-GGAGAAGCCTTTGTATGGGGTGTGCTTCACACCAGAGAGCTCCGCATCACCCTGTG
as: 5'-CACAGGGTGATGCGGAGCTCTCTGGTGTGAAGCACACCCCATACAAAGGCTTCTCC
myomaker-3'UTR-mut2:
s: 5'-CCTGTGCCAGAGAGCTCCGCATCACTTCACACGCCCTTGGCCACTTCACCTCCCCC
as: 5'-GGGGGAGGTGAAGTGGCCAAGGGCGTGTGAAGTGATGCGGAGCTCTCTGGCACAGG
Myomaker 0.6kb promoter-mut1:
s: 5'-GTGGCTCAGCTGTGCCACTGCCTGTGAAGATTCCCTGCCTGCAGCTCTCCGGGC
as: 5'-GCCCGGAGAGCTGCAGGCAGGGAATCTTCACAGGCAGTGGCACAGCTGAGCCAC
Myomaker 0.6kb promoter-mut2:
s: 5'-AAGAAGGAATGGAGCATATGGGTGGCTACTTGTTGCCACTGCCTGCAGCTGTTCCCTGC
as: 5'-GCAGGGAACAGCTGCAGGCAGTGGCAACAAGTAGCCACCCATATGCTCCATTCCTTCTT

For RACE and gene specific PCR the following primers were used:
5' RACE RT-adapter: 5' -GGCCACGCGTCGACTAGTACTTTTTTTTTTTTTTTTT
5' outer primer: 5' -AAGCAGTGGTATCAACGCAGAGTACGCGGGGGGGGGG
5' inner primer: 5'-AAGCAGTGGTATCAACGCAGAGT
5' Myomaker outer primer: 5'- TGTCTGGATACAGCCCTTTC
5' Myomaker inner primer: 5'- TGAAACATAGCACCGATAAGC

3'RACE Oligo(dT)-anchor primer:
5'-GCTGTCAACGATACGCTACGTAACGGCATGACAGTGTTTTTTTTTTTTTTTTT
3' adaptor outer primer: 5'- GCTGTCAACGATACGCTACGTAACG
3' adaptor inner primer: 5'- CGCTACGTAACGGCATGACAGTG
3' Myomaker outer primer: 5'- TGGCTTATCGGTGCTATGTT
3' Myomaker inner primer: 5'- CCCACCAGTTCCATACGC
